# The influence of teacher–student relationship on Chinese high school students' academic motivation for the ideological and political subject: the mediating role of academic emotions

**DOI:** 10.3389/fpsyg.2023.1329439

**Published:** 2024-01-08

**Authors:** Yufeng Wang, Guohai Jiang, Zhendong Yao, Lei Liu

**Affiliations:** ^1^School of Public Administration, Hunan Normal University, Changsha, China; ^2^College of Marxism, Hunan Normal University, Changsha, China; ^3^Normal College, Hunan University of Arts and Science, Changde, China; ^4^Public Administration School, Guangzhou University, Guangzhou, China

**Keywords:** teacher–student relationship, academic emotions, academic motivation, China, high school students, the ideological and political subject

## Abstract

**Introduction:**

This study investigated the influence of teacher–student relationship on Chinese high students' academic motivation for the ideological and political subject and the parallel mediating roles of positive and negative academic emotions on this influence.

**Method:**

The participants of this study were 425 Chinese high school students. This study measured teacher–student relationship, academic motivation, and academic emotions through several self-reported questionnaires. Structural equation models were used to analyze data and investigate the direct and indirect influence of teacher–student relationship on Chinese high school students' academic motivation for the ideological and political subject.

**Results:**

Teacher–student relationship had a significant, positive, and direct impact on Chinese high school students' academic motivation for the ideological and political subject. Moreover, positive and negative academic emotions, in parallel, mediated the relationship between these two factors.

**Discussion:**

This study demonstrates the important influence of teacher–student relationships on Chinese high school students' academic motivation for the ideological and political subject. A positive teacher–student relationship can directly increase students' academic motivation for this subject and indirectly enhance their motivation by generating positive academic emotions. Therefore, teachers should express care for their students, make friends with them, and be their partners in learning and life. Additionally, teachers need to pay close attention to students' academic emotions and provide them emotional support so that they can develop positive academic emotions while learning, and strive to establish and maintain a good teacher–student relationship.

## 1 Introduction

The ideological and political subject in Chinese high schools is a comprehensive subject that aims to cultivate students' sense of political identity, scientific spirit, civic consciousness, and legal awareness; educate students on ideology and morality; and improve their ability to participate in public affairs. This subject has been taught in Chinese high schools for a long time and is an important compulsory subject for most students. Chinese high school students must pass the academic-level exam for this subject to obtain a high school diploma, and for students who choose this subject as one of their college entrance exams, their grade in this subject accounts for a large proportion of their total score in the college entrance exam.

The subject is greatly valued in the Chinese high school education system. However, it is highly theoretical, comprising content such as ideological, political, philosophical, and moral theories, which are uninteresting, abstract, and difficult to understand for high school students and are removed from their daily lives. Moreover, given that students need to pass the exam and obtain high scores, they have to memorize a lot of content that they find boring. Therefore, most Chinese high school students consider this subject tedious, useless, and unappealing and believe that memorizing its content takes a lot of time and effort. This negative impression of the ideological and political subject can lead students to become bored when studying the subject, distract their learning attention, and weaken their motivation to learn it. Thus, students need powerful motivation to encourage them to study the subject, which is important to increase their knowledge and improve their performance in exams. Research has shown that a good classroom environment can increase students' engagement in learning activities and motivate them to work hard (Velayutham and Aldridge, 2013). As an essential component of the classroom environment, the teacher–student relationship has been proven to be crucial in enhancing students' academic motivation (Hughes, 2012).

Many researchers have confirmed that teacher–student relationship is an important factor that influences students' learning behavior and academic results (Gest et al., 2005). Students' academic motivation (Yunus et al., 2011) and development of prosocial behavior (Longobardi et al., 2021) are strongly influenced by teacher–student relationship. Teachers ought to make an effort to establish good relationships with their students in their studies and daily lives, rather than just imparting knowledge, and teachers and students should respect, trust, and understand each other (Mokhele, 2006). It is currently unclear how teacher–student relationship affects students' academic motivation for the ideological and political subject in Chinese high schools, especially in the context of traditional Chinese Confucian culture. Therefore, to clarify this matter, this study investigates the influence of teacher–student relationship on students' academic motivation for the aforementioned subject and the mediating role of academic emotions (both positive and negative). It provides targeted guidance for motivating high school students to study the ideological and political subject actively.

## 2 Literature review

### 2.1 Teacher–student relationship and academic motivation

Teacher–student relationship is an interpersonal relationship that gradually develops during communication and interactions between teachers and students. As one of the most critical and fundamental interpersonal relationships in schools, it significantly affects adolescents' academic development and psychological health (Lippard et al., 2018). Teacher–student relationship mainly manifests through emotional, cognitive, and behavioral communication. Teacher–student relationship has three different levels—a specific working relationship, a natural interpersonal relationship, and a profound social relationship—which constitute the real sense of interpersonal relationships in an actual society (Dong and Liu, 2002). Teacher–student relationship has a significant impact on students' academic performance and success (Roorda et al., 2011). A harmonious and supportive teacher–student relationship motivates students to learn and improves academic performance (Barile et al., 2012).

Confucius, the most famous educator, and philosopher in Chinese history, placed great importance on the harmony of the teacher–student relationship in his educational philosophy. In his first Chinese pedagogical work, written more than 2,000 years ago, he pointed out that only when students are close to and respect their teachers can they believe in and learn from the knowledge and truths taught by them. China has been deeply influenced by the traditional Confucian culture from ancient times to the present day. The traditional Confucian culture emphasizes that students must respect teachers and their authority, and there is the notion in the traditional Confucian culture that once a teacher, always a father. On the one hand, the traditional Confucian culture stresses the harmonious, cordial and family-like relationship between teachers and students, which is conducive to building a good teacher-student relationship. On the other hand, the traditional Confucian culture places great emphasis on students' respect for teachers' authority, and students may be afraid to communicate with teachers due to fear of their authority, which can have a negative impact on teacher-student relationship.

A supportive student–teacher relationship can offer students a bond with their school and high-quality learning resources. Youths are more likely to achieve better academic outcomes when they have a close and harmonious relationship with their teachers and are glad to accept their guidance. In the absence of support and guidance from their teachers, students will have difficulty accessing the resources for their academic success (Ma et al., 2022). If students view their teachers as “partners” in learning, they will have a stronger motivation to learn and achieve better academic performance. A high-quality teacher–student relationship can eliminate fear and stress in students' minds, facilitate the development of a common language, promote mutual understanding between both parties, reduce the generation gap, and create a good learning environment. When teachers report closer relationships with students, in turn, students demonstrate modestly stronger outcomes across all domains. In contrast, more conflictual relationships are largely associated with underachievement (Ansari et al., 2020). Several teacher practices are capable of meeting adolescents' developmental needs and, as such, are likely to positively influence adolescents' developmental and academic trajectories (McHugh et al., 2013).

If the teacher–student relationship is close and warm, students will study harder, engage in more challenging academic activities, perform better in class, and meet or exceed their teachers' expectations in the learning process. By contrast, a low-quality teacher–student relationship will worsen the learning environment for students, which can hinder communication between students and teachers and even deepen misunderstandings and prejudices, thus reducing students' effort, patience, confidence, and motivation. Teachers' interpersonal behavior is crucial for the quality of the student–teacher relationship. The research results indicated that there is a significant difference between teachers' self–perceptions and students' perceptions of teacher interpersonal behavior. The differences among teachers' and students' perceptions can partially be explained by teachers' years of teaching experience and the degree level, as well as the class educational level (Karamane et al., 2023).

Academic motivation is a crucial concept in education. As a key variable in the field of education and training, both educationalists and psychologists perceive academic motivation as an essential factor in students' academic performance and psychological health. Academic motivation is defined as a student's desire to learn or interest in learning (Hulleman et al., 2016). It is an important driving force for students to strive to learn. Academic motivation is associated with academic participation, educational attainment, academic success, and psychological health (Froiland et al., 2012). Australian education scholar John Biggs divided academic motivation into three categories: deep motivation, surface motivation, and achievement motivation. Deep motivation refers to students' motivation to learn because of their inherent interest in the learning content. Surface motivation refers to the student's motivation to cope with and pass exams. Achievement motivation refers to students' motivation to gain higher grades, rewards, and praise. Existing research suggests that academic motivation has a vital influence on the achievement of academic success (Dogan, 2015). The efforts and activities of each student in the process of achieving academic success depend on the quality of their academic motivation. A high level of academic motivation promotes students' interest, patience, and effort in learning (Nancy, 2009). Students with higher academic motivation usually have a stronger initiative to learn. Hence, these students more probably would study harder and achieve better academic results. Classrooms can be seen as miniature but complete societies. Teachers' attitudes and behaviors toward students and how they interact with students may directly or indirectly impact students' academic motivation (Hughes, 2011).

Research has confirmed that teacher–student relationship has a significant effect on students' academic motivation. Social psychological studies on teacher–student relationships suggest that students who perceive their relationships with teachers as positive, warm, and intimate have a stronger motivation to improve their academic results. A high-quality teacher–student relationship is beneficial for students to develop stronger academic motivation (O'Connor and McCartney, 2007). A positive teacher–student relationship is conducive to creating a warm, supportive, and harmonious classroom environment, which helps students learn more actively and communicate with teachers more proactively (Birch and Ladd, 1997). Additionally, some researchers claim that the degree to which students believe that their teachers take care of them also affects their academic motivation. The more students believe their teachers take care of them, the more motivated they are to learn (Murdock and Miller, 2003). In addition, if students like their teachers, the school environment, and all other school members, they will study more actively, which will positively impact their learning participation and learning process (Skipper and Douglas, 2015). For example, when students like their teachers, they usually have a stronger motivation to learn in the classroom, hoping to gain their teachers' attention and affection through good performance and achievement. Conversely, when students dislike their teachers, they usually lack the motivation to learn and even hate the subject taught by the teacher, which can significantly reduce their academic performance.

Many theories on motivation and participation provide useful frameworks for studying the link between teacher–student relationship and academic motivation in educational settings. Social motivation theory suggests that if students feel social support from their teachers, they will develop strong motivational beliefs in the learning process, which will motivate them to engage actively in learning activities and achieve good academic results (Furrer and Skinner, 2003). According to self-determination theory, individuals have three basic psychological needs: autonomy, competence, and relatedness (Ryan and Deci, 2000). If these basic psychological needs are met, people will be more motivated, active, and happy to work and learn, which would promote psychological health and effective participation. Specifically, autonomy as motivation is central to self-determination theory. In classroom situations, a supportive teacher–student relationship is conducive to forming independent motivation in students, which could enhance their learning autonomy and promote their active learning behavior (Korthagen et al., 2014; McNally and Slutsky, 2018). For example, students who are in an autonomous and supportive learning environment have stronger intrinsic motivation and are more proactive and attentive to their learning.

Bowlby (1982) attachment theory generally holds that infants who view their parents as providing a safe foundation can explore with less fear and distractions. If their parents are around, infants will have greater courage and confidence to explore more. Attachment theory suggests that a supportive teacher–student relationship can offer a safe foundation for students to gain knowledge, develop abilities, achieve aspirations, and pursue new goals; reduce their worries and distractions in learning; motivate them to learn more actively and attentively; and encourage them to complete more challenging learning tasks, and thus improve their academic achievements (Hughes et al., 2008; Ma et al., 2018). Meanwhile, expectancy-value theory suggests that students' academic participation is determined by their expectations of achieving academic success and their perceptions of the value or importance of their learning. When students believe that they can achieve academic success, they may become more actively engaged in learning and put effort into their studies. Students who doubt that they can achieve academic success are less likely to participate actively in learning or invest a lot of energy, especially when they encounter difficulties in learning tasks or negative feedback. If students feel that what they are learning is important, they will be more active and patient when learning. As important socializers in schools, teachers can significantly influence students' expectations and values. A supportive teacher–student relationship is beneficial for students to generate positive expectations and value in learning, which further promotes their engagement in learning, and, in turn, greatly stimulates their academic motivation and enhances their academic achievements.

Teacher–student interaction and student participation jointly determine students' academic performance, and teacher–student relationship has an important impact on students' academic motivation, learning behavior, and academic performance. A warm and positive teacher–student relationship can stimulate students' enthusiasm for learning, enhance their interest and academic motivation, and develop their advantages (Fredrickson and Losada, 2005; Daniel et al., 2016). Students with positive, warm, and intimate relationships with teachers usually have positive attitudes toward learning in the classroom. They are also more engaged in what they are learning, cope better with stress, accept their teachers' guidance and criticism more seriously, and pay more attention to teachers than others in learning (Ferradás et al., 2017). Additionally, these students are more likely to persevere when they complete difficult tasks (Koca, 2016). By contrast, a negative teacher–student relationship negatively impacts learning activities and the students themselves, thus weakening their motivation to learn and leading to difficulties in concentrating on their studies, which could result in academic failure (Lei et al., 2023). Students with poor relationships with teachers generally lack academic motivation, are reluctant to exert effort, are more likely to give up when faced with difficult learning tasks, and rarely communicate and interact with their teachers.

### 2.2 The mediating role of academic emotions

Academic emotions refer to the emotions related to teaching or learning that students experience in the learning process (Pekrun et al., 2002). Control-value theory (Pekrun, 2006) holds that academic emotions are generated under various learning conditions, classes, and examinations. Researchers have generally categorized students' academic emotions into two types: positive and negative academic emotions. Positive academic emotions include happiness, confidence, enjoyment, excitement, relaxation, and pride; negative academic emotions include anxiety, distress, anger, boredom, hopelessness, and inferiority. These positive and negative academic emotions can affect students' academic motivation and impact their academic performance. Researchers have shown that students are more likely to spend time and energy on learning activities that are relaxing, fun, and enjoyable, rather than those that cause tension, distress, or boredom (Frenzel et al., 2007). Positive academic emotions can motivate students to study hard, help increase their attention to learning, contribute to the development of their cognitive activities, and make them creative, which, in turn, helps in enhancing their academic performance (Yu and Dong, 2005). By contrast, negative academic emotions are thought to reduce students' academic motivation, hinder their learning and cognition development, and decrease their attention to learning, thereby leading to failure in academic performance (Fredrickson, 2004).

Many studies have shown a significant relationship among teacher–student relationship, academic emotions, and academic motivation. A good teacher–student relationship can produce an emotional connection for students. Such emotional connections make students feel confident and comfortable in the presence of their teachers and peers, which contributes to their academic success. A positive and intimate teacher–student relationship can activate positive emotions related to learning, increase satisfaction with psychological needs in school, and reduce academic stress and burnout among students (Furrer et al., 2014; Clem et al., 2021). A higher quality of teacher–student relationship was associated with stronger positive and weaker negative academic emotions. Students who felt cared about, respected, and understood by their teachers were more motivated to engage in interactions, discussions, and learning. Moreover, academic emotions helped induce and maintain students' interest in learning activities (Krapp, 2005). When teachers have good relationships with students, students are more likely to develop positive academic emotions such as relaxation, confidence, and pleasure in the classroom, which can enhance their academic motivation. By contrast, a negative teacher–student relationship can lead to students developing negative academic emotions such as tension, inferiority, and pain in the learning process, which can weaken students' motivation to learn. Studies have demonstrated that positive academic emotions are related to higher academic motivation levels and negative academic emotions are related to lower academic motivation levels (Trigwell et al., 2012). As mentioned above, academic emotions may mediate the relationship between teacher–student relationship and academic motivation.

## 3 The present study

The current study explored the influence of teacher–student relationship on students' academic motivation for the ideological and political subject in Chinese high schools and the mediating role of academic emotions on this influence. Two dimensions of academic emotions, positive and negative academic emotions, were included in the analysis model, and their roles were compared. The guiding hypotheses for this study are as follows:

Hypothesis 1: Teacher–student relationship has a significant positive direct effect on students' academic motivation for the ideological and political subject in Chinese high schools.
Hypothesis 2: Positive and negative academic emotions act in parallel to mediate the influence of teacher–student relationship on students' academic motivation for the ideological and political subject in Chinese high schools.

## 4 Materials and methods

### 4.1 Participants

The participants were 425 students from high schools in mainland China, comprising 219 men (51.53%) and 206 women (48.47%); 142 were in first grade (33.41%), 158 in second grade (37.18%), and 125 in third grade (29.41%), with an average age of 17.08 years (SD = 0.89 range 16–19 years).

### 4.2 Measures

#### 4.2.1 Teacher–student relationship evaluation scale

This study used the teacher–student relationship evaluation scale developed by Chu (2006) to measure teacher–student relationship in high school in China. The scale has three dimensions. Namely, the teacher–student relationship situation, teachers' affinity, and differences in status between teachers and students. Each dimension has six items and each item is rated on a five-point Likert scale, ranging from 1 to 5. The higher (lower) the score, the better (worse) the teacher–student relationship. The CFA results indicated that the scale has a good construct validity (x^2^/df = 1.153, CFI = 0.994, TLI = 0.993, RMSEA = 0.019, SRMR = 0.028), The Cronbach's alpha coefficient for the entire scale in this study was 0.83, indicating good reliability.

#### 4.2.2 High school students' academic emotional scale

The high school students' academic emotional scale used in this study was adapted from the relevant dimensions of the Adolescent Academic Emotion Questionnaire, originally developed by (Dong and Yu, 2007). The aforementioned scale comprises two subscales, namely, the positive and negative academic emotions scales. This study measured Chinese high school students' academic emotions using 36 questions on a five-point scale ranging from 1 (*complete nonconformity*) to 5 (*complete conformity*), and the higher the score, the higher the level of related traits. In this study, the Cronbach's alpha coefficient of the sub-scales of positive and negative academic emotion were 0.81 and 0.86, respectively.

#### 4.2.3 Academic motivation scale

The academic motivation scale was adapted from the study process questionnaire, developed by British psychologist (Biggs, 1987). This scale consists of three dimensions: surface motivation, deep motivation, and achievement motivation, and has 18 items to assess Chinese high school students' academic motivation for the ideological and political subject. Each item is rated on a five-point Likert scale, ranging from 1 (*never*) to 5 (*often*), with higher scores representing greater academic motivation. This scale has good reliability and validity. In this study, Cronbach's alpha coefficient for this scale was 0.78.

### 4.3 Data analysis

#### 4.3.1 Common method bias test

The exploratory factor analysis results showed that 21 factors had eigenvalues >1. The explained variance percentage of the first factor was 24.76%, which is far lower than the 40% recommended by Podsakoff et al. (2003), indicating that method bias was not an issue in this study.

#### 4.3.2 Preliminary analysis

The results of the descriptive statistics and correlational analysis of the relationships among teacher–student relationship, academic emotions, and students' academic motivation for the ideological and political subject in Chinese high schools are presented in Table 1. Teacher–student relationship was directly, positively, and significantly related to the students' academic motivation for the ideological and political subject (*r* = 0.62, *p* < 0.001) and to positive academic emotions (*r* = 0.51, *p* < 0.001). By contrast, teacher–student relationship was directly, negatively, and significantly related to negative academic emotions (*r* = −0.48, *p* < 0.001). Positive academic emotions were directly, positively, and significantly related to the students' academic motivation for the ideological and political subject (*r* = 0.60, *p* < 0.001), whereas negative academic emotions were directly, negatively, and significantly related to the students' academic motivation for the ideological and political subject (*r* = −0.57, *p* < 0.001; see Table 1).

**Table 1:** Means, standard deviations, and correlations among the main variables.

	**M**	**SD**	**1**	**2**	**3**	**4**
1. TSR	3.07	0.70	1			
2. PAE	3.24	0.66	0.51^**^	1		
3. NAE	2.94	0.60	−0.48^**^	−0.67^**^	1	
4. AMIPS	3.02	0.64	0.62^**^	0.60^**^	−0.57^**^	1

TSR, teacher-student relationship; PAE, positive academic emotion; NAE, negative academic emotion; AMIPS, academic motivation for the ideological and political subject. ^**^P **<** 0.01.

#### 4.3.3 Measurement model

By measuring the extent of the model's fit, we found that the intermediary effect model and the data fit well (see Table 2).

**Table 2:** Model fit statistics for the structural equation modeling.

**Model**	**X^2^/df**	**CFI**	**TLI**	**RMSEA (90%)**
Teacher–student relationship and academic motivation for the ideological and political subject	3.16	0.98	0.97	0.072 (0.059, 0.085)

CFI, comparative fit index; TLI, Tucker–Lewis index; RMSEA, root mean square error of approximation.

#### 4.3.4 Mediating variables

**Figure 1:**
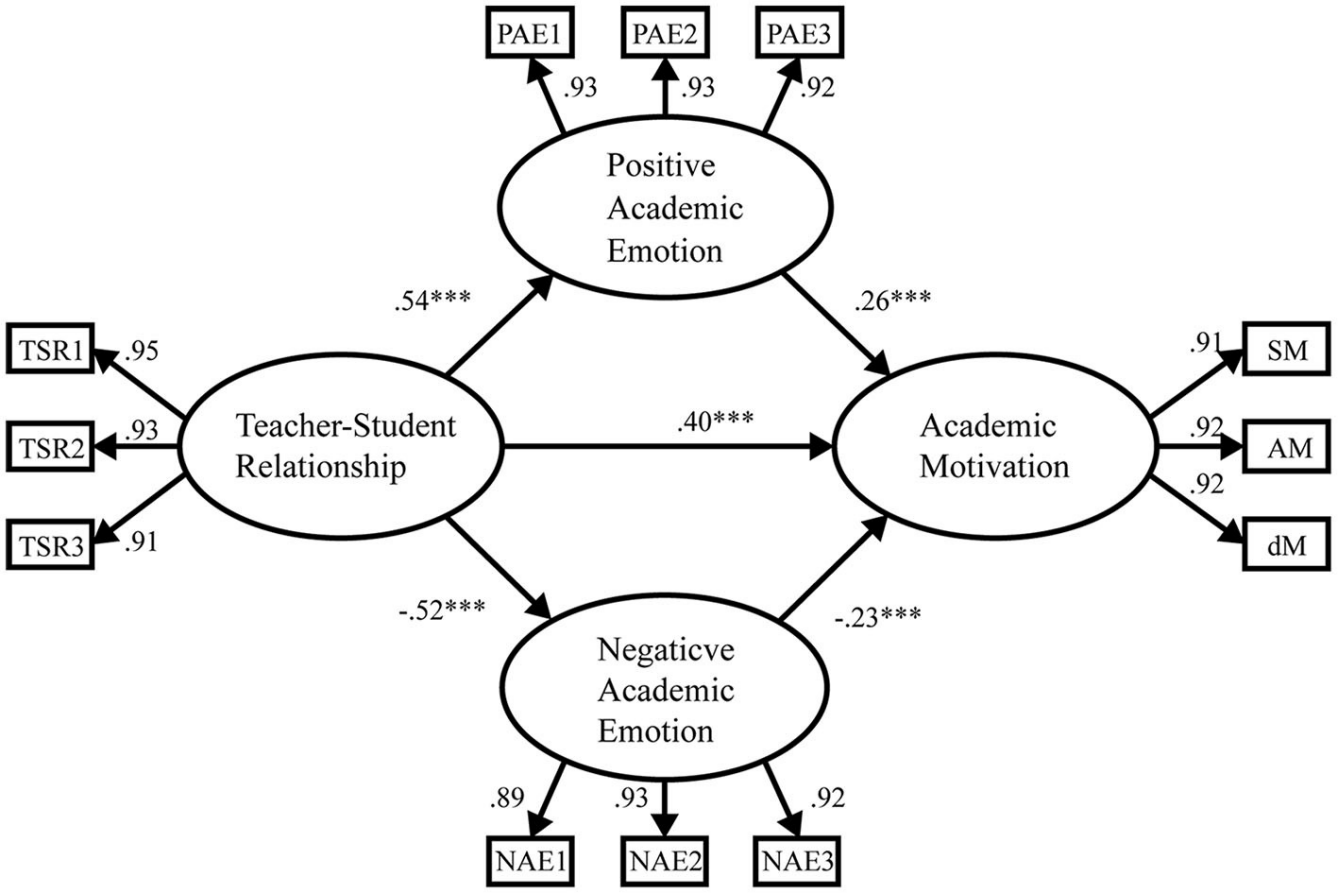
Standardized path coefficients for the proposed model. TSR1-3, three parcels of teacher–student relationship; PAE1-3, three parcels of positive academic emotions; NAE1-3, three parcels of negative academic emotions; SM, surface motivation; AM, achievement motivation; and DM, deep motivation; ^***^P < 0.001.

We used SEM to examine the mediating effects of positive and negative academic emotions on teacher–student relationship and students' academic motivation for the ideological and political subject. The mediation model in Figure 1 shows that teacher–student relationship had a significant positive direct effect on students' academic motivation for the ideological and political subject (β = 0.40, *t* = 8.67, *p* < 0.001), thus confirming Hypotheses 1. Moreover, teacher–student relationship had a significant positive direct effect on positive academic emotions (β = 0.54, *t* =11.98, *p* < 0.001). Meanwhile, positive academic emotions had a significant positive direct effect on students' academic motivation for the ideological and political subject (β = 0.26, *t* = 4.78, *p* < 0.001). Teacher–student relationship had a significant negative direct effect on academic emotions (β = −0.52, *t* = −11.33, *p* < 0.001), and negative academic emotions had a significant negative direct effect on students' academic motivation for the ideological and political subject (β = −0.23, *t* = −4.33, *p* < 0.001).

We also performed a bias-corrected bootstrapping analysis based on 5,000 resamples with 95% confidence intervals to examine the significance of the mediating effects of teacher–student relationship on students' academic motivation for the ideological and political subject. The results indicated that the mediating effect of positive and negative academic emotions on teacher–student relationship and students' academic motivation for the ideological and political subject was significant (see Table 3). Positive and negative academic emotions play a parallel mediating role between teacher–student relationship and the students' academic motivation for the ideological and political subject, thus confirming Hypothesis 2.

**Table 3:** Bootstrap analysis of the mediating model.

**Effect**	**Path**	**Standardized β**	**The size of the effect**	**95%CL**
Total	TSR → AMIPS	0.66		
Direct	TSR → AMIPS	0.40		
Indirect	TSR → PAM → AMIPS	0.14	21%	(0.072, 0.178)
Indirect	TSR → NEM → AMIPS	0.12	18%	(0.057, 0.161)

TSR, teacher–student relationship; PAE, positive academic emotion; NAE, negative academic emotion; and AMIPS, academic motivation for the ideological and political subject.

## 5 Discussion

The current study explored the influence of teacher–student relationship on students' academic motivation for the ideological and political subject in Chinese high schools, and the parallel mediating roles of positive and negative academic emotions on this influence. First, the study results demonstrated that teacher–student relationship directly, positively, and significantly influenced Chinese high school students' academic motivation for the ideological and political subject. Students with good (poor) relationships with their teachers tended to have a stronger (weaker) academic motivation for the subject. A positive teacher–student relationship can increase students' interest in the content of the ideological and political subject, create a good classroom environment for them, and improve their enthusiasm for classroom learning, which, in turn, enhances their academic motivation for the subject. By contrast, a negative teacher–student relationship will increase students' aversion and fear toward their teachers, hinder communication, and interaction between both parties, and weaken their interest and initiative in studying the ideological and political subject, thus decreasing their academic motivation for the subject.

Consistent with previous studies, the findings indicated that teacher–student relationship can predict students' academic motivation for the focal subject. A positive teacher–student relationship can strengthen students' motivation and interest in learning, provide space for them to share opinions, and further stimulate them to participate actively in learning activities (Verschueren and Koomen, 2012). If students get along well with their teachers, they would have a greater sense of security and freedom and be more motivated to learn (Urdan and Schoenfelder, 2006). Establishing and maintaining a positive teacher–student relationship can improve students' patience and willingness to learn, make them actively solve any problems encountered during the learning process, promote successful adaptation in school, and improve their academic performance in class (Griffin et al., 2013). By contrast, a negative teacher–student relationship can reduce students' interest, enthusiasm, and motivation for learning. If teachers often criticize students, shout at them, or are too strict with them, students would communicate less with their teachers and be unlikely to study hard (Opdenakker et al., 2012; Mensah and Eric Koomson, 2020). The findings of this study indicated that the better the teacher–student relationship, the stronger the students' academic motivation for the ideological and political subject in Chinese high schools.

Second, the outcomes of the current study indicated that positive academic emotions mediated the relationship between teacher–student relationship and students' academic motivation for the ideological and political subject. Specifically, if the teacher–student relationship is warm and close, the students would have a greater likelihood of developing positive academic emotions such as relaxation, confidence, pleasure, and pride in the process of learning the ideological and political subject. In addition, positive academic emotions can simultaneously enhance students' academic motivation for the aforementioned subject in Chinese high schools. When students have positive academic emotions while studying this subject, they will be more proactive, conscientious, persevering, and interested in learning.

Many previous studies have similar results to the present study. A positive teacher–student relationship has a major influence on students' academic emotions and is vital to all students. A higher-quality teacher–student relationship is related to a higher level of wellbeing and a lower level of anxiety (Capern and Hammond, 2014; Mainhard et al., 2018). The higher the teacher–student relationship quality, the more likely students will have positive learning experiences. If the teacher–student relationship is supportive, students would perceive the teacher as warm and approachable in the classroom environment, and experience pleasant emotions (Martin, 2009; Becker et al., 2014). Furthermore, positive emotions can enhance academic motivation (Kim and Hodges, 2012). For example, positive academic emotions can induce and maintain students' interest in learning materials, stimulate their academic motivation, and promote more active participation in learning (Ainley et al., 2005; Kahu, 2013). Based on these analyses, positive academic emotions mediated the relationship between teacher–student relationship and students' academic motivation for the subject.

Finally, the study results demonstrated that negative academic emotions mediated the relationship between teacher–student relationship and students' academic motivation for the ideological and political subject. Specifically, teacher–student relationship was negatively related to students' negative academic emotions toward the said subject. If teacher–student relationship is negative, students will produce negative academic emotions such as anxiety, pain, boredom, and inferiority while learning the subject. Meanwhile, negative academic emotions will reduce students' academic motivation for the subject. Students with negative academic emotions usually have lower academic motivation and interest in learning, are more likely to give up when facing difficult learning tasks, are easily distracted in the learning process, and are reluctant to put in time and effort to study this subject.

These findings align with those of previous studies. Long-term teacher–student conflicts may lead to the accumulation of negative academic emotions over time. Negative teacher–student relationship damage students' self-esteem and make them feel inferior (Miller et al., 2000; Buyse et al., 2008). If students perceive that their teachers are punishing them, they will generate negative emotions such as anxiety and nervousness (Frenzel et al., 2007). Negative academic emotions undermine students' interest in learning, cause them to reduce their learning efforts, and lead to lower overall academic performance (Bibby, 2002). Furthermore, these negative academic emotions inhibit students' cognitive and physiological processes, thereby decreasing their learning initiative and motivation (Clore and Huntsinger, 2009; Pekrun et al., 2017). Moreover, negative academic emotions can lead to negative self-evaluation and loss of confidence in pursuing academic success among students, which reduce their academic motivation and academic performance. Our study provides new evidence for the effect of negative academic emotions in the association between teacher–student relationship and students' academic motivation for the subject.

## 6 Conclusions

This study revealed the influence of teacher–student relationship on students' academic motivation for the ideological and political subject in Chinese high schools and the mediating role of positive and negative academic emotions. The study findings clarified the mechanisms by which teacher–student relationship affects students' academic motivation for the ideological and political subject, thus providing targeted guidance for improving students' academic motivation for the subject. Our findings showed that teacher–student relationship had a direct, positive, and significant influence on students' academic motivation for the ideological and political subject. Therefore, teachers should express care for their students, make friends with them, be their partners in learning and life, and create a good learning environment for students in the classroom.

Furthermore, this study found that positive and negative academic emotions, in parallel, mediated the relationship between teacher–student relationship and Chinese high school students' academic motivation for the ideological and political subject. Teacher–student relationship can indirectly influence students' academic motivation for the subject through academic emotions (both positive and negative). Therefore, teachers should pay close attention to students' emotions; express enthusiasm, warmth, concern, and love to students in various ways; provide emotional support for students so that they can generate positive academic emotions such as pleasure, confidence, relaxation, and happiness in the process of learning the ideological and political subject; and work to establish and maintain a good teacher–student relationship with them.

## 7 Research limitations and future study

This study achieved the expected goals and obtained satisfactory results; however, there are some limitations that must be noted. Firstly, the study only surveyed participants in two provinces of mainland China, and the sample size was relatively small, therefore, it may not be possible to generalize the research findings to high school students from other cultural backgrounds or geographical regions. In further studies, the regional scope and sample size of the survey should be broadened to enhance generalization. Secondly, the data for this study were collected from self-reports, and the results of self-report measures may be influenced by individual subjectivity, which may have an adverse impact on the reliability of the research findings. Future research should consider improving the objectivity of data resources through more diverse data collection channels. Third, this study was a cross-sectional study with limited causal assumptions. In future studies, a longitudinal survey should be conducted to investigate causal relationships.

## Data availability statement

The raw data supporting the conclusions of this article will be made available by the authors, without undue reservation.

## Ethics statement

The studies involving humans were approved by Academic Ethics Committee of Hunan Normal University. The studies were conducted in accordance with the local legislation and institutional requirements. Written informed consent for participation in this study was provided by the participants' legal guardians/next of kin.

## Author contributions

YW: Conceptualization, Data curation, Resources, Software, Writing—original draft. GJ: Methodology, Supervision, Validation, Writing—review & editing. ZY: Formal analysis, Investigation, Writing— review & editing. LL: Project administration, Visualization, Writing—review & editing.
